# Intensity-modulated proton radiotherapy spares musculoskeletal structures in regional nodal irradiation for breast cancer: a dosimetric comparison

**DOI:** 10.2340/1651-226X.2024.40084

**Published:** 2024-10-01

**Authors:** Jessica F. Burlile, Satomi Shiraishi, Heather J. Gunn, Jennifer L. Bradt, Haley M. Kroeplin, Karen G. Lang, Jenna K. Cimmiyotti, Nicolas Depauw, Connie Y. Chang, Kevin M. Brom, Cassandra L. Sonnicksen, Anhmai Vu, Rachel B. Jimenez, Kimberly S. Corbin

**Affiliations:** a Mayo Clinic Department of Radiation Oncology, Rochester, MN, USA; b Mayo Clinic Department of Medical Physics, Rochester, MN, USA; c Mayo Clinic Department of Quantitative Health Sciences, Rochester, MN, USA; d Mayo Clinic Department of Rehabilitation, Rochester, MN, USA; e Massachusetts General Hospital Department of Radiation Oncology, Boston, MA, USA; f Massachusetts General Hospital Department of Radiology, Boston, MA, USA; g Mayo Clinic Alix School of Medicine, Rochester, MN, USA

**Keywords:** Proton therapy, breast cancer, arm dysfunction, cancer survivorship, cancer rehabilitation, chronic toxicity

## Abstract

**Background and purpose:**

Regional nodal irradiation (RNI) for breast cancer delivers radiation in proximity to the shoulder and torso, and radiation exposure may contribute to long-term upper extremity and postural morbidity. To date, no studies have assessed the differential dosimetric impact of proton versus photon radiation on shoulder and torso anatomy. This study examined clinically relevant musculoskeletal (MSK) structures and assessed the dose delivered with each modality.

**Patients/material and methods:**

Ten MSK structures were contoured on IMPT (intensity-modulated proton therapy) and VMAT (volumetric modulated arc therapy) plans for 30 patients receiving RNI. Relevant dose metrics were compared for each of the structures. Intensity-modulated proton therapy dose was calculated using the relative biological effective value of 1.1. Hypo-fractionated plans were scaled to the equivalent dose in 2 Gy fractions (EQD2) using an alpha/beta ratio of four. Wilcoxon signed rank sum tests compared doses. Select three-dimensional and optimised VMAT plans were also informally compared.

**Results and interpretation:**

Each of the 10 structures received a statistically significantly lower dose with the use of IMPT compared with VMAT. Differences were greatest for posterior structures, including the trapezius, latissimus dorsi and glenohumeral joint. Mean absolute differences were as great as 23 Gy (supraspinatus D5cc) and up to 30-fold dose reductions were observed (deltoid D50cc). An average 3.7-fold relative dose reduction existed across all structures. Measures of low/intermediate dose (V15Gy and D50cc) showed the largest differences.

Intensity-modulated proton therapy results in statistically lower radiation exposure to relevant shoulder and torso anatomy compared to photon radiation for patients requiring RNI. Prospective study is needed to correlate functional outcomes with radiation dose.

## Introduction

Breast cancer is the most commonly diagnosed cancer in the United States (US), and nearly 4 million people today are breast cancer survivors [1, 2]. With increases in both the incidence of breast cancer and therapeutic advancements, the prevalence of survivors is expected to increase [3]. As such, greater numbers of people may face long-lasting toxicities from cancer-directed therapy, heightening the importance of characterising and reducing these toxicities in order to maximise quality of life.

Upper extremity dysfunction is common after breast cancer treatment, especially among those requiring multi-modality therapy. In one study, 65% of patients who underwent axillary lymph node dissection (ALND), comprehensive regional nodal irradiation (RNI) and chemotherapy had upper extremity dysfunction within 2 years of completing treatment, with 44% reportedly sacrificing activities they enjoyed as a result [4]. Lymphedema, joint and muscle fibrosis, brachial plexopathy and peripheral neuropathy may all contribute to dysfunction. Overhead actions require normal biomechanics of the shoulder girdle, and decreased tissue mobility and activation of structures of the rotator cuff can lead to pain and limited motion [5, 6]. Furthermore, asymmetry of the shoulder and trunk musculature after surgery and radiation may lead to significant postural changes [5].

While ALND is considered the primary risk factor for long-term upper extremity dysfunction [6], RNI further increases this risk [7, 8]. Few studies have investigated the impact of radiation dose on the musculoskeletal (MSK) structures of the arm, shoulder and back [9], and a better understanding of a dose-response relationship could enable radiation plan optimisation for improved functional outcomes. The use of proton therapy may result in lower radiation exposure to posterior MSK structures, which may have functional implications for patients who require RNI.

In this study, we examined the radiation dose distribution to the intrinsic muscles and joints of the shoulder among patients receiving RNI for breast cancer. We hypothesised that using proton radiation would result in significantly reduced doses to the MSK structures implicated in upper extremity and postural dysfunction.

## Patients/materials and methods

Thirty patients who received either proton (pencil beam scanning intensity modulated proton therapy, IMPT) or photon (volumetric modulated arc therapy, VMAT) comprehensive nodal irradiation at Mayo Clinic or Massachusetts General Hospital between 2018 and 2023 were included. These patients had comparison plans generated so that both a photon and proton plan were available in the treatment planning software. This study was approved by the Mayo Clinic and Massachusetts General Hospital institutional review boards.

Ten MSK structures of interest were identified in collaboration with an oncologic specialised physical therapist and an MSK radiologist. The structures of interest were selected based on their contribution to upper extremity dysfunction and truncal asymmetry after breast cancer treatments. A summary of the muscular structures and their function can be found in Table 1. Joint and bony structures included the acromioclavicular (AC) joint, glenohumeral (GH) joint and humeral head. Muscles included the deltoid, latissimus dorsi, pectoralis major, serratus anterior, supraspinatus and trapezius. A rotator cuff attachments (RCA) structure was created by expanding the humeral head contour by 1 cm and cropping the distal portion of the structure within the humeral head contour (See the Supplemental Materials for contouring details). Each of these 10 structures were contoured for 30 patients by two radiation oncologists (JB, RJ) and a total of 60 radiation plans (30 proton and 30 photon) were analysed. Review and modification of individual contour sets by the two contouring physicians were performed to ensure consistency across the data set.

**Table 1:** Seven muscular structures and their functions.

Anatomical Movement	Deltoid	Latissimus	Pectoralis	Trapezius	Serratus Anterior	Supraspinatus	Rotator Cuff
Scapula elevation				x			
Scapula depression		x	x	Lower			
Scapula up-rotation				x	x		
Scapula down-rotation		x	x				
Scapula protraction			x		x		
Scapula retraction		x		x			
Arm flexion	Ant		Clav				
Arm extension	Post	x	Sternal				x
Arm abduction	x					x	x
Arm adduction		x	x				x
Internal rotation	Clav	x	x				x
External rotation	Post						x

Ant: anterior portion of the deltoid; Clav: clavicular portion of the deltoid or pectoralis;; Lower: lower portion of the trapezius muscle; Post: posterior portion of the deltoid.

Clinically relevant dose metrics were selected for comparison between the proton and photon plans. For small volume structures (AC joint, GH joint, humeral head, and RCA), the mean dose, V15 Gy and V30 Gy were evaluated. For large structures and those for which most of the structural volume would lie outside of the treatment field, we compared the D5cc, D10cc and D50cc. These same dose metrics were utilised for the pectoralis major because of its larger size, despite the fact that a portion of this muscle often lies within the target volume.

These dose metrics were chosen to reflect previously published literature regarding dose-response relationships and MSK dysfunction. Namely, sarcoma and head and neck literature correlates high doses (>50 Gy) with MSK dysfunction [10, 11] and one publication within the breast literature reports more MSK dysfunction when a larger area of the shoulder receives 15 Gy [9]. Therefore, we included V15 Gy and V30 Gy to assess medium-dose levels and D5 cc and D10 cc metrics to capture high doses.

The lowest dose clinical target volume (CTV) was the whole breast or chest wall, levels 1–3 of the axilla, supraclavicular fossa and ipsilateral internal mammary lymph nodes (IMN). Areas of boost included the lumpectomy cavity, chest wall, incision or IMN. For the lowest dose CTV, all plans utilised prescription doses of 40.05–50 Gy in 15–25 once daily fractions (2.0–2.67 Gy per fraction). Boosts were delivered sequentially or were integrated, for a total high dose CTV ranging from 48 to 60 Gy. Dose scaling was performed prior to analysis to allow direct comparisons between dose and fractionation schemes across plans.

Plans were created using the Eclipse treatment planning system, version 15.1 (Varian Medical Systems, Palo Alto, California) or Astroid, version 1.3 (Massachusetts General Hospital, Boston, MA). A typical pencil beam scanning proton plan consisted of one to three anterior, anterolateral or posterolateral beams, and VMAT plans consisted of three to four partial arcs. Partial arcs were 6 MV energy, co-planar and moved between an optimal medial tangent angle to a posterior angle. The first and second arcs used 5–20 degree complementary collimator angles. Because of the larger field lengths necessary to cover the planning target volume (PTV), the third and fourth arcs were split into upper and lower arcs and collimated to 90 degrees to maximise the travel of the x-jaw. The third and fourth arc overlapped by 2–4 cm at isocenter for each patient. For proton plans, radiation dose was calculated using a relative biological effective (RBE) value of 1.1 [12, 13]. Proton energies varies based on the depth of each layer in the pencil beam scanning volume, meaning that each plan utilised protons of multiple energies. Both proton and photon plans were optimised so that either CTV (proton) or PTV (photon) target coverage was D90% ≥90% (priority 1) and D95% ≥95% (priority 2). Boost volumes shared those same target objectives.

To collect dose metrics for our research query, differential dose volume histograms (DVH) were calculated and the dose was scaled to the equivalent dose in 2 Gy fractions (EQD2) using an alpha/beta ratio of four (for muscle) [14] with a Matlab code. Thereafter, cumulative DVH for EQD2 were calculated for plotting, and metrics were extracted. Wilcoxon signed rank sum tests were executed to compare the proton and VMAT plan doses on the individual patient level, for the selected metrics. To account for multiplicity given that nearly 30 comparisons were performed after evaluating three dose metrics for each of the 10 MSK structures (the D50cc metric was not compared for the supraspinatus because of its small size <50cc), we applied the Bonferroni correction. Thus, for all 29 comparisons, two-sided α = 0.00172 was used to determine statistical significance.

In a secondary analysis, 15 three-dimensional (3D) plans were available for dose comparison, and 12 VMAT plans were re-optimised to intentionally lower dose to the 10 MSK structures of interest. Dose constraints for the MSK structures were determined by examining the un-optimised VMAT doses and the IMPT doses, with objectives ranging from 60 to 80% of un-optimised VMAT doses (unless this resulted in a number less than IMPT doses, in which case the objective was 80–100% of the un-optimised VMAT dose). Coverage of the PTV remained the highest priority, and sparing of standard OAR (organs at risk) such as the heart and lungs was a higher priority than sparing the MSK structures. For these optimised VMAT plans and 3D plans, dose data were extracted and scaled to EQD2 using a Matlab code similarly to the 30 VMAT and IMPT plans. A statistical comparison of the 15 IMPT, un-optimised VMAT, and 3D plans is presented in the Supplemental materials. The Wilcoxon signed rank sum test was utilised for this comparison and the same α = 0.0172 was used for significance after applying the Bonferroni correction.

## Results

### Plan and patient characteristics

Of the 30 patients evaluated, 4 underwent lumpectomy while the remainder had mastectomy. Six patients received a boost as part of their radiation plan, and most were simultaneously integrated. Most plans were conventionally fractionated (50 Gy in 25 fractions), although four were moderately hypo-fractionated (40.05 Gy in 15 fractions). Twenty of 26 mastectomy surgeries were followed by reconstructive procedures: 9 patients received up-front permanent implants and 11 had tissue expanders placed. See Table 2 for more detailed plan and treatment characteristics.

**Table 2:** Characteristics of regional nodal irradiation plans and patient treatments.

Treatment	Number	Percent of entire cohort*	Total per treatment	Percent of treatment**
Primary surgery				
Lumpectomy	4	13		NA
Mastectomy	26	87	30	NA
Reconstruction post-mastectomy				
Elected reconstruction	20	67		77
No reconstruction	6	20	26	23
Reconstruction at time of radiation				
Tissue expander	11	37		55
Implant in place	9	30	20	45
Implant type				
Sub-pectoral	7	23		78
Pre-pectoral	2	7	9	22
Radiation fractionation				
Conventional	26	87		NA
Moderate hypofractionation	4	13	30	NA
Radiation boost				
Yes	6	20		NA
No	24	80	30	NA
Boost technique				
Simultaneously integrated	4	13		67
Sequential	2	7	6	33
Boost area				
Chest wall or skin	4	13		67
Internal mammary	1	3		17
Lumpectomy cavity	1	3	6	17
Institution				
Institution 1	20	67		NA
Institution 2	10	33	30	NA

* Percent of Entire Cohort may not equal 100% if this treatment did not apply to the entire cohort; i.e. reconstruction does not apply to those who underwent lumpectomy.

** Percent of Treatment will always equal 100% and refers to the sub-group in the first column. NA: not applicable, if the Percent of Treatment is the same as Percent of Entire Cohort.

### Dosimetry

With the use of IMPT, each structure received a statistically significantly lower dose at the majority of the queried dose metrics, including posterior, anterior and midline structures. A mean 3.7-fold relative dose reduction existed across all structures. Posterior structures tended to benefit most from the utilisation of IMPT. For example, the supraspinatus D5cc was 23.3 Gy less with protons (a 4.1-fold dose reduction), and the latissimus D50cc was 18.5 Gy less when using protons (3.0-fold dose reduction). D50cc for the trapezius was nearly five-fold less when using protons. Complete dose reduction data for each structure and each metric is shown in Table 3.

**Table 3:** Selected dose metrics for clinically relevant MSK structures.

Structure	Metric	Mean IMPT dose	Mean VMAT dose	Absolute difference between means	Proton relative dose reduction	*p* *
Deltoid	D5 cc	10.4 Gy	24.9 Gy	14.5 Gy	2.39	<0.001*
	D10 cc	4.8 Gy	21.6 Gy	16.8 Gy	4.5	<0.001*
	D50 cc	0.2 Gy	6.2 Gy	6.0 Gy	31	<0.001*
Latissimus dorsi	D5 cc	43.8 Gy	48.2 Gy	4.4 Gy	1.1	<0.001*
	D10 cc	38.6 Gy	45.7 Gy	7.1 Gy	1.18	<0.001*
	D50 cc	9.5 Gy	28 Gy	18.5 Gy	2.95	<0.001*
Serratus anterior	D5 cc	47.3 Gy	50.0 Gy	2.7 Gy	1.06	<0.001*
	D10 cc	43.8 Gy	47.0 Gy	3.2 Gy	1.07	0.001*
	D50 cc	17.1 Gy	28.7 Gy	11.6 Gy	1.68	<0.001*
Supraspinatus	D5 cc	7.5 Gy	30.8 Gy	23.3 Gy	4.11	<0.001*
	D10 cc	5.6 Gy	27.7 Gy	22.1 Gy	4.95	<0.001*
Trapezius	D5 cc	18.9 Gy	30.4 Gy	11.5 Gy	1.61	<0.001*
	D10 cc	13.6 Gy	26.2 Gy	12.6 Gy	1.93	<0.001*
	D50 cc	3.1 Gy	14.8 Gy	11.8 Gy	4.77	<0.001*
Pectoralis major	D5cc	52.1 Gy	54.2 Gy	2.2 Gy	1.04	<0.001*
	D10cc	51.7 Gy	53.8 Gy	2.2 Gy	1.04	<0.001*
	D50cc	49.3 Gy	51.2 Gy	1.9 Gy	1.04	<0.001*
Acromioclavicular joint	Mean	9.5 Gy	16.0 Gy	6.4 Gy	1.68	<0.001*
	V15 Gy	23.10%	41.80%	18.70%	1.81	0.001*
	V30 Gy	18.80%	17.30%	-1.50%	0.92	0.641
Glenohumeral joint	Mean	7.9 Gy	26.3 Gy	18.43 Gy	3.33	<0.001*
	V15 Gy	19.70%	86.80%	67.10%	4.41	<0.001*
	V30 Gy	2.60%	38.60%	36.00%	14.85	<0.001*
Humeral head	Mean	5.1 Gy	12.6 Gy	7.5 Gy	2.47	<0.001*
	V15 Gy	11.80%	33.40%	21.60%	2.83	<0.001*
	V30 Gy	2.80%	7.70%	4.90%	2.75	0.021
Rotator cuff attachments	Mean	7.4 Gy	15.3 Gy	7.9 Gy	2.07	<0.001*
	V15 Gy	19.00%	40.60%	21.60%	2.14	<0.001*
	V30 Gy	8.60%	16.60%	8.00%	1.93	<0.001*

* Indicates statistical significance at an alpha = 0.00172 given the Bonferroni correction for multiple comparisons. MSK: musculoskeletal; IMPT: intensity-modulated proton therapy; VMAT: volumetric modulated arc therapy.

Midline structures such as the GH joint also showed a significantly greater absolute percentage difference in mean dose: V15 Gy was an absolute 67.1% lower with the use of protons, equating to a 4.4-fold reduction in the area receiving 15 Gy. The GH joint V30 Gy was an absolute 36.0% lower with protons (14.6-fold area reduction). The RCA structure V15 Gy metric was an absolute 21.6% lower with the use of protons (2.1-fold area reduction), and all three humeral head metrics (mean, V15 Gy and V30 Gy) were two to three-fold lower in proton plans.

In general, anterior structures exhibited smaller dose difference between IMPT and VMAT. However, the mean deltoid D5cc (highest doses within the anterior head of the deltoid) was 14.5 Gy lower by using IMPT (a 2.4-fold reduction). Doses to the pectoralis major were relatively similar between proton and photon plans, and absolute reduction was only approximately 2 Gy for the D5cc, D10cc, and D50cc metrics – but lower for IMPT plans.

**Figure 1:**
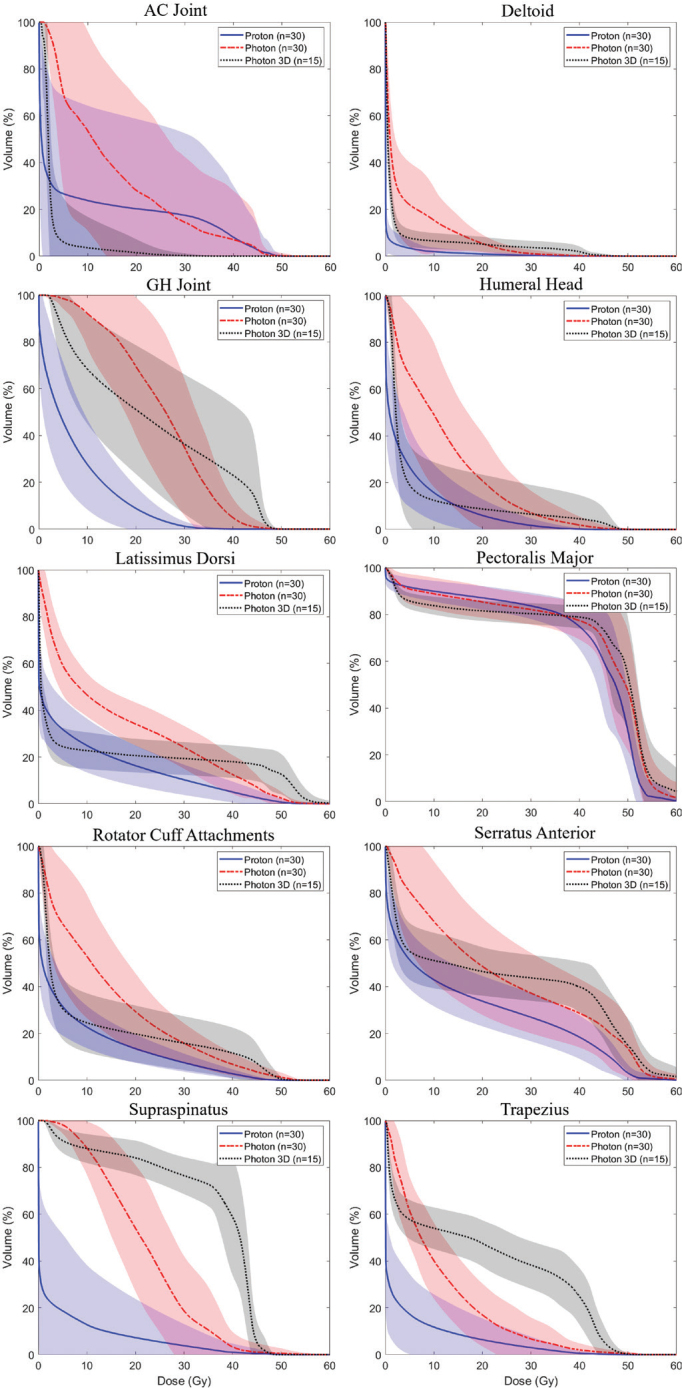
Dose volume histograms for each of the 10 MSK structures. Proton plans are represented in blue, VMAT plans are red, and 3D plans are black. The solid or dashed primary line represents the mean DVH for each structure and modality, while the shaded area surrounding the primary lines represents one standard deviation from the mean. The integral dose is lower for many structures with the use of IMPT, although 3D radiation does a superior job sparing the AC joint. Note that *n* = 30 for IMPT and VMAT plans while *n* = 15 for 3D plans. MSK: musculoskeletal; IMPT: intensity-modulated proton therapy; VMAT: volumetric modulated arc therapy; DVH: dose volume histograms; AC: acromioclavicular.

**Figure 2:**
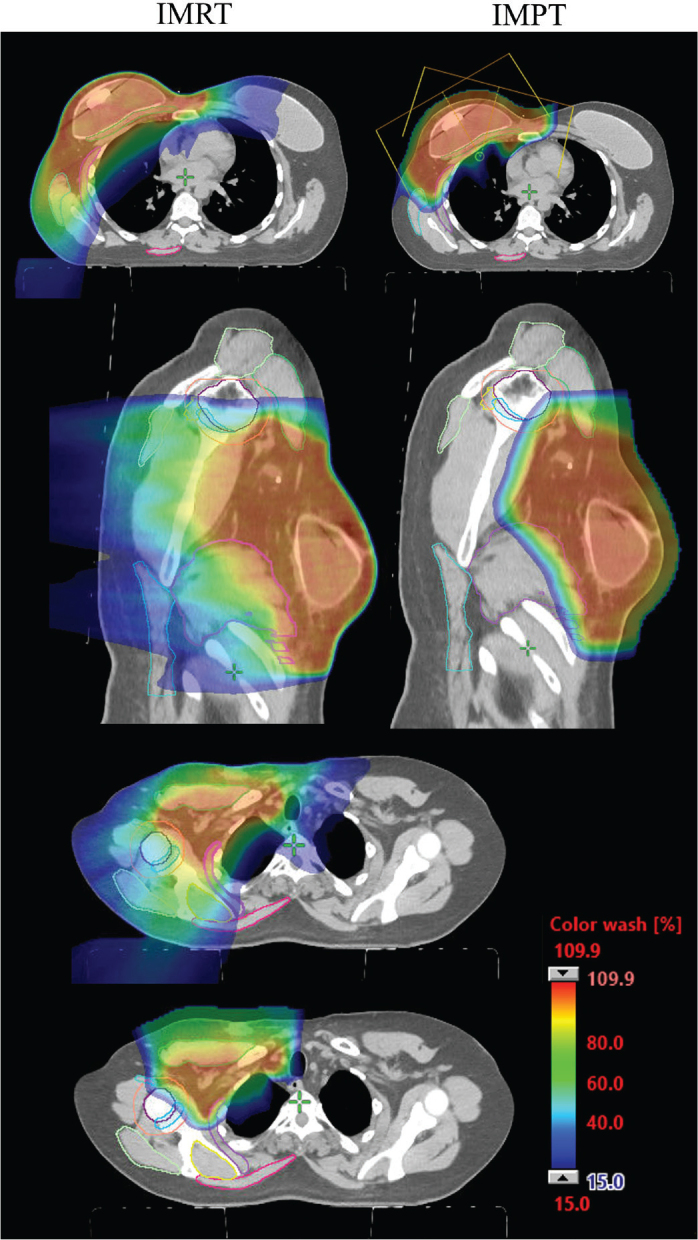
Example VMAT and IMPT plan for a patient who is status-post mastectomy and tissue expander reconstruction. Note the anterior beam arrangement in the IMPT plan in the first axial image. Posterior MSK structures such as the latissimus dorsi, trapezius, supraspinatus, and posterior portions of the serratus anterior, humeral head, rotator cuff attachments, and deltoid are notably spared. MSK: musculoskeletal; IMPT: intensity-modulated proton therapy; VMAT: volumetric modulated arc therapy.

The median relative dose reduction for the three posterior structures (supraspinatus, latissimus dorsi and trapezius), four midline structures (GH joint, AC joint, RCA and humeral head), and three anterior structures (deltoid, pectoralis major and serratus anterior) was a 2.44-fold, 2.31-fold and 1.07-fold reduction, respectively. Aggregate DVH curves (Figure 1) demonstrate a lower integral dose with the use of IMPT, although degrees of difference varied both with individual patient anatomy and institutional radiation planning practices. Figure 2 shows an example patient’s proton and photon RNI plan, emphasising the sparing of posterior structures through anterior proton beam arrangement.

### 3D plan comparison

Three-dimensional radiation plans were available for 15 of the patients in this study. In general, posterior MSK structures (supraspinatus, latissimus dorsi and trapezius) were better spared by VMAT. Midline, superolateral structures (GH joint, AC joint, RCA and humeral head) were more effectively spared with 3D radiation.

Anterior structures (deltoid, pectoralis major and serratus anterior) received similar doses with 3D radiation and VMAT. The D5 cc metric was on average slightly higher with 3D while the D50 cc metric was on average slightly lower with 3D. See Table 4 for 3D dose data, and Supplemental Table 1 for the results of the Wilcoxon signed rank sum test comparing 3D radiation doses to both unoptimised VMAT and IMPT doses.

**Table 4:** Comparing IMPT, VMAT, optimised VMAT, and 3D plans.

Structure	Metric	IMPT (*n* = 30)	VMAT (*n* = 30)	Optimised VMAT (*n* = 12)	3D (*n* = 15)
Deltoid	D5 cc	10.4 Gy	24.9 Gy	20.0 Gy	32.3 Gy
	D10 cc	4.8 Gy	21.6 Gy	17.3 Gy	22.7 Gy
	D50 cc	0.2 Gy	6.2 Gy	4.1 Gy	1.5 Gy
Latissimus dorsi	D5 cc	43.8 Gy	48.2 Gy	48.3 Gy	53.1 Gy
	D10 cc	38.6 Gy	45.7 Gy	44.6 Gy	52.2 Gy
	D50 cc	9.5 Gy	28 Gy	23.9 Gy	15.9 Gy
Serratus anterior	D5 cc	47.3 Gy	50.0 Gy	52.6 Gy	53.4 Gy
	D10 cc	43.8 Gy	47.0 Gy	50.9 Gy	52.4 Gy
	D50 cc	17.1 Gy	28.7 Gy	31.2 Gy	30.1 Gy
Supraspinatus	D5 cc	7.5 Gy	30.8 Gy	27.0 Gy	43.4 Gy
	D10 cc	5.6 Gy	27.7 Gy	22.2 Gy	42.7 Gy
Trapezius	D5 cc	18.9 Gy	30.4 Gy	25.2 Gy	44.4 Gy
	D10 cc	13.6 Gy	26.2 Gy	20.5 Gy	43.3 Gy
	D50 cc	3.1 Gy	14.8 Gy	9.1 Gy	29.7 Gy
Pectoralis major	D5cc	52.1 Gy	54.2 Gy	53.9 Gy	55.7 Gy
	D10cc	51.7 Gy	53.8 Gy	53.4 Gy	55.0 Gy
	D50cc	49.3 Gy	51.2 Gy	50.7 Gy	52.1 Gy
Acromioclavicular joint	Mean	9.5 Gy	16.0 Gy	5.8 Gy	2.6 Gy
	V15 Gy	23.1%	41.8%	6.7%	2.5%
	V30 Gy	18.8%	17.3%	0.0%	0.2%
Glenohumeral joint	Mean	7.9 Gy	26.3 Gy	19.3 Gy	22.9 Gy
	V15 Gy	19.7%	86.8%	70.5%	58.9%
	V30 Gy	2.6%	38.6%	9.8%	36.3%
Humeral head	Mean	5.1 Gy	12.6 Gy	9.2 Gy	5.9 Gy
	V15 Gy	11.8%	33.4%	20.6%	10.2%
	V30 Gy	2.8%	7.7%	3.4%	6.5%
Rotator cuff attachments	Mean	7.4 Gy	15.3 Gy	12.4 Gy	10.2 Gy
	V15 Gy	19.0%	40.6%	31.3%	21.9%
	V30 Gy	8.6%	16.6%	10.9%	15.8%

IMPT: intensity-modulated proton therapy; VMAT: volumetric modulated arc therapy.

### Optimised VMAT plans

After examining the doses achieved by VMAT and IMPT, 12 VMAT plans were re-optimised in an attempt to further lower doses to the 10 MSK structures of interest. With the exception of the latissimus dorsi D5 cc metric and all serratus anterior metrics, optimised VMAT plans achieved lower MSK structure doses, on average, than un-optimised VMAT plans. For the superolateral GH joint, humeral head, and RCA structure, optimising VMAT plans achieved doses intermediate to VMAT (highest) and 3D (lower) doses, but did not achieve doses as low as IMPT. With rare exceptions (AC joint metrics, humeral head V15 Gy), IMPT delivered less dose than VMAT, optimised VMAT or 3D radiation plans. (See Table 4 for mean optimised VMAT plan doses.)

## Discussion

A comparison of dose to clinically relevant MSK structures showed a clear dosimetric advantage to using IMPT over VMAT for RNI. In general, the largest dose reductions were seen for metrics which measured low to moderate doses over larger areas (such as the V15 Gy and D50 cc metrics) and for posterior and midline structures (trapezius, latissimus dorsi, supraspinatus and GH joint). Anterior structures such as the pectoralis major and serratus anterior showed a smaller difference in dose between IMPT and VMAT plans, which is consistent with the physical properties of protons and an anterior beam arrangement.

While select adverse outcomes after breast radiotherapy have been thoroughly investigated, including cardiopulmonary disease, lymphedema and reconstructive outcomes [15, 16], MSK dysfunction has received considerably less attention. Despite the potential effects on activities of daily living, occupation and hobbies, there remains a paucity of data examining the effects of breast cancer therapy on specific upper extremity and thoracic anatomy. Surgery remains the strongest predictor of upper extremity dysfunction for patients with breast cancer [17], but radiation-related soft tissue changes, which typically develop and progress in the months to years after therapy, may contribute to the chronicity of dysfunction [18]. This problem is most pronounced in the setting of combined ALND and RNI, and in the absence of diligent physical therapy or exercise [19, 20].

Research regarding radiation-induced muscle dysfunction, including a dose-response relationship, is sparce. In other disease sites, including sarcoma and head and neck cancer, doses exceeding 50 Gy have been shown to contribute to MSK dysfunction [10, 11]. Although not as well studied, lower dose exposure, particularly in conjunction with surgery, may be important for function after breast cancer treatment. For example, up to 60% of patients with breast cancer experience reduced shoulder flexion and abduction at 1 month after surgery [21], and in a patient-reported outcomes analysis of the SENOMAC trial, 12 and 33% of patients (sentinel lymph node biopsy and ALND, respectively) reported moderate or severe arm dysfunction at 1 year after surgery [22]. Nearly all patients on this study received RNI. In the AMAROS trial, arm function worsened after surgery and improved over the course of 5 years, but never returned to baseline [23].

The present study focusses on 10 MSK structures which are correlated with some of the most commonly observed MSK difficulties after breast cancer treatments: reduced arm abduction, flexion and internal rotation [24], frozen shoulder (adhesive capsulitis) [25], difficulty maintaining symmetric thorax posture [5] and the development of myofascial pain and trigger points [26].

The supraspinatus and deltoid muscles are important for arm abduction, and minimising small areas of high dose (D5cc, D10cc) can be effectively achieved by utilising IMPT. The trapezius, latissimus dorsi, serratus anterior and pectoralis major can all develop active myofascial trigger points after surgery and radiation [27]. The trapezius experienced a dose reduction of 11–13 Gy in the D5cc, D10cc, and D50cc distributions with the use of proton therapy. These reductions in higher doses over smaller areas may translate to an improvement in function, but further study is needed.

The humeral head, GH joint and AC joint are involved in the development of adhesive capsulitis and frozen shoulder, which may affect up to 10% of patients after surgery [25]. The GH and AC joint capsules, as well as RCAs, work synergistically for proper biomechanical positioning and changes in the length and tension of those connective tissues can result in shoulder pathology [28]. The lower doses delivered with IMPT may help prevent these soft tissue changes and reduce the chronicity of upper extremity functional limitation.

Prior reports examining 3D radiation techniques and possible effects on shoulder dose and patient-reported function are noteworthy. Specifically, intensity modulated radiation therapy (IMRT) has been shown to reduce dose to a ‘shoulder’ OAR when compared to 3D radiation planning [29]. While that study did not delineate specific muscles or joint structures, patient scores on the q-DASH shoulder and arm assessment tool (Quick-Disabilities of the Arm, Shoulder, and Hand questionnaire) tended to be better in patients treated with IMRT compared to 3D radiation; which did correlate with lower doses [29]. Johansen et al. correlated upper extremity dysfunction with a larger volume of a ‘shoulder’ OAR receiving 15 Gy, highlighting the importance of low to intermediate doses to MSK structures [9]. All patients in this study received 3D radiation.

When comparing 3D and VMAT dose metrics in the present study, it is evident that VMAT plans delivered less dose at the D5 cc and D10 cc levels. 3D radiation tended to consistently deliver less dose at the D50 cc level. Notably, utilising 3D radiation lowered the V15 Gy percentage beyond what was achievable by un-optimised VMAT, and for superior and lateral structures such as the AC joint, GH joint, humeral head and RCA, doses approached those achieved by IMPT. The ability to block these superolateral OARs and the absence of the low- to medium dose wash inherent to VMAT likely explains these differences. For the AC joint, 3D radiation delivered less dose than IMPT – the only structure for which this was true.

Optimising VMAT plans effectively lowered doses for almost every MSK OAR (with the exception of the serratus anterior), and resulted in doses closer to, but not reaching or surpassing, IMPT plans. It is clear that a better understanding of which components of the shoulder and trunk musculature contribute most to MSK dysfunction is critical as the dose metrics from IMPT, VMAT and 3D plans are compared. As we gain additional information about which structures are most important for function, optimising IMPT or VMAT plans to avoid these structures has the potential to improve functional outcomes.

This study reports structure-specific dose data from VMAT and IMPT radiation plans, laying the foundation for an improved understanding of the relationship between dose and functional outcomes. This study has several limitations, the first of which is the absence of functional and patient-reported outcomes to correlate with these structure-specific dose differences. Additionally, we did not have access to 3D radiation plans for all patients, limiting our analysis of this data. Only a subset of VMAT plans could be re-optimised, limiting formal statistical dose comparison to unoptimised plans. Finally, IMPT plans were not re-optimised to avoid MSK structures, but given the success of optimising VMAT plans, this represents an interesting avenue of future study.

Intensity-modulated proton therapy reduces dose to multiple functionally relevant shoulder and back muscles, joints and bony structures. This study is the first to examine specific shoulder structures in the context of comparing dose distribution between proton and photon RNI plans. While IMPT represents an opportunity for dose reduction, prospective study of the relationship between dose sparing and functional outcomes is needed.

## Author contributions

JFB: Data curation, investigation, visualisation, writing (original draft), writing (reviewing and editing).

HMK, KGL, JKC: Data curation, investigation, methodology, writing (reviewing and editing).

RBJ: Data curation, investigation, writing (reviewing and editing), conceptualisation, methodology, supervision.

KSC: Writing (reviewing and editing), conceptualisation, methodology, supervision.

SS: Visualisation, writing (reviewing and editing), methodology, formal analysis.

HJG: Writing (reviewing and editing), methodology, formal analysis.

ND: Data curation, writing (reviewing and editing), formal analysis.

CYC: Writing (reviewing and editing), conceptualisation.

AV: Writing (reviewing and editing), visualisation.

KMB: Data curation.

CLS: Data curation.

JLB: Data Curation, writing (reviewing and editing), conceptualisation, supervision.

## Supplementary Material

Intensity-modulated proton radiotherapy spares musculoskeletal structures in regional nodal irradiation for breast cancer: a dosimetric comparison

## Data Availability

For original data, please contact the corresponding author at burlile.jessica@mayo.edu.
