# Supplementary material for: Intensity-modulated proton radiotherapy spares musculoskeletal structures in regional nodal irradiation for breast cancer: a dosimetric comparison
Source: Acta Oncol. 2024 Oct 1;63:40084. doi: 10.2340/1651-226X.2024.40084 (PMC11538476; doi:10.2340/1651-226X.2024.40084)
Supplement: Intensity-modulated proton radiotherapy spares musculoskeletal structures in regional nodal irradiation for breast cancer: a dosimetric comparison [file AO-63-40084-s1.pdf]

Supplementary material has been published as submitted. It has not been copyedited, or typeset by Acta Oncologica

Supplemental Materials

Contouring guide

General principles: When contouring muscles that extend inferior to the CTV, contour up to 3 cm inferior to the CTV. In general, the latissimus generally extends far inferior to the CTV and the serratus anterior may extend inferior to the CTV by a smaller amount. Note in the sagittal CT image below how the posterior cyan/blue colored latissimus extends 3 cm inferior to the red anterior CTV before the contour ceases. The purple mid-image serratus anterior also extends <1 cm inferior to the CTV in this patient's plan, although this is a bit harder to appreciate.

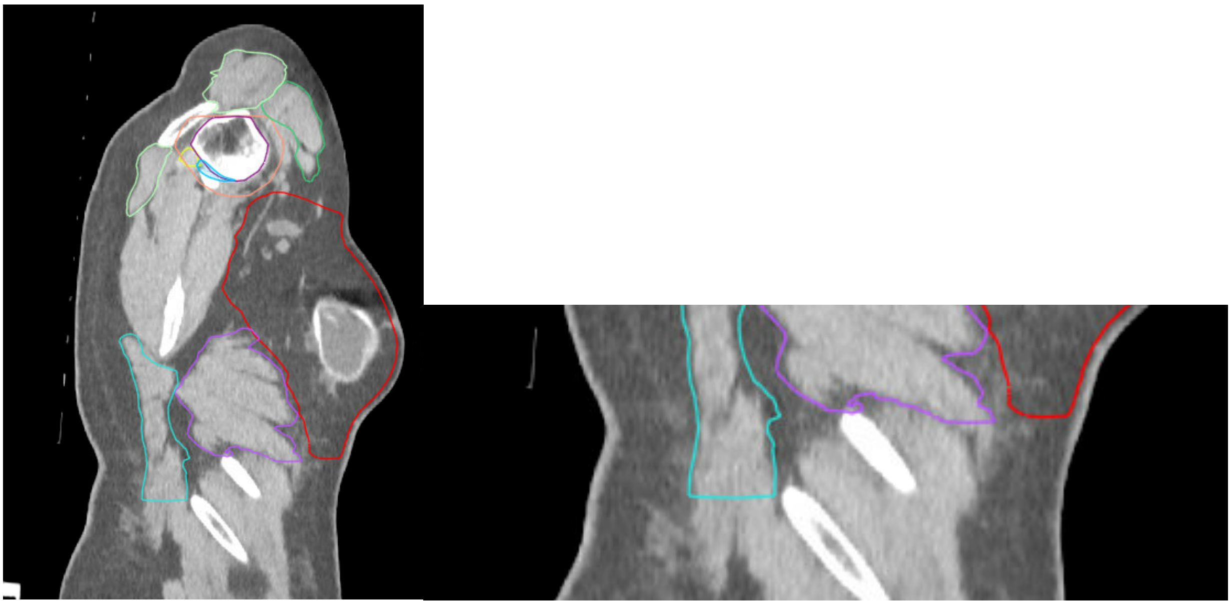

Guidelines for contouring specific structures in alphabetical order: the CTV appears red in all images, for reference.

1) Acromioclavicular Joint (light purple in all images): Use the bone window to better visualize the joint. Use a 1 cm brush centered in the space between the clavicle and the acromion. Contour all slices for which the space between the bones is 1 cm or less. The second image shows a larger separation at the end of the structure.

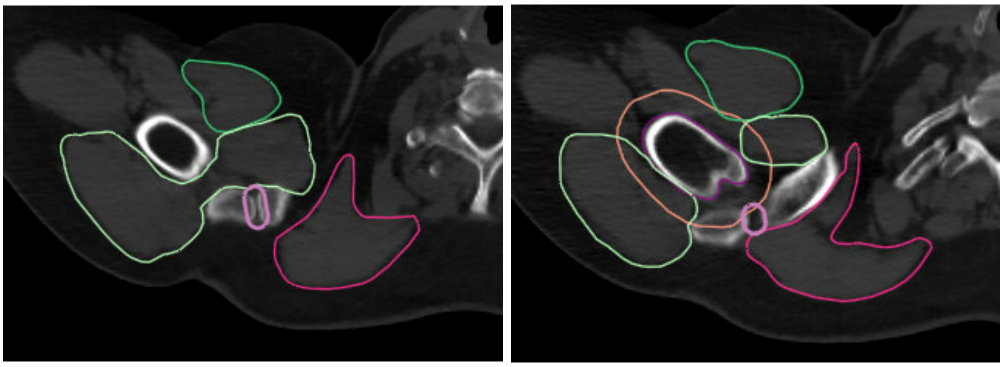

2) Deltoid (pale green in all images): Contour the posterior, lateral, and anterior deltoid as one structure. If the patient's arms are up, this structure will begin superiorly at the deltoid tuberosity of the humerus. It originates at the clavicle and two areas of the scapula (acromion process and spine of the scapula).

The first image shows the deltoid insertion at the deltoid tuberosity of the humerus. Traveling inferiorly the pectoralis major (kelly green) and trapezius (bright pink) come into view, as well.

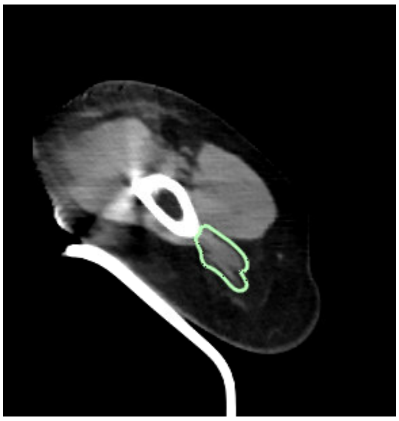

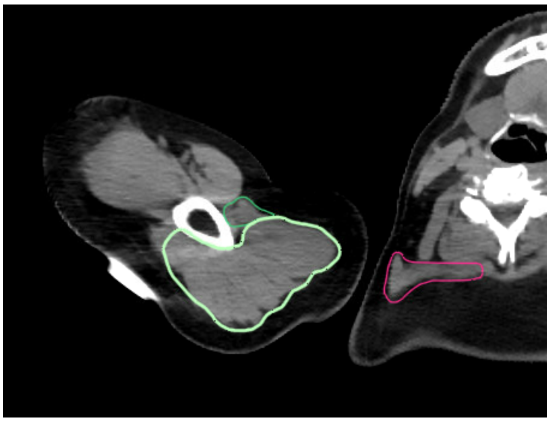

With arms up, traveling superiorly to inferiorly, the contour will at one point be split into two separate contours as the glenohumeral joint comes into view and the anterior and posterior deltoid continue more inferiorly. See the two images below.

Also seen below, the lateral deltoid begins to disappear at its origin at the acromian process of the scapula. The anterior and posterior deltoid continue as two separate contours as the scan continues inferiorly. Also seen at this level is the kelly green pectoralis major, the orange rotator cuff attachment structure, and the bright pink trapezius.

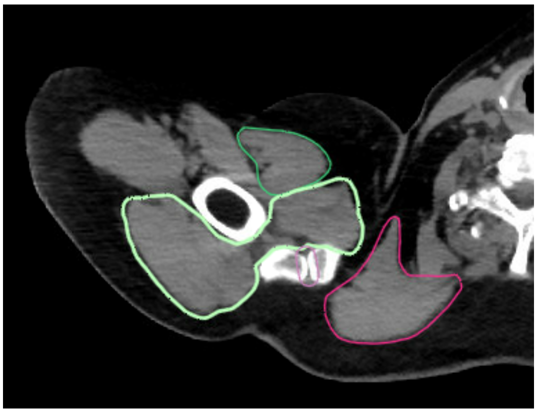

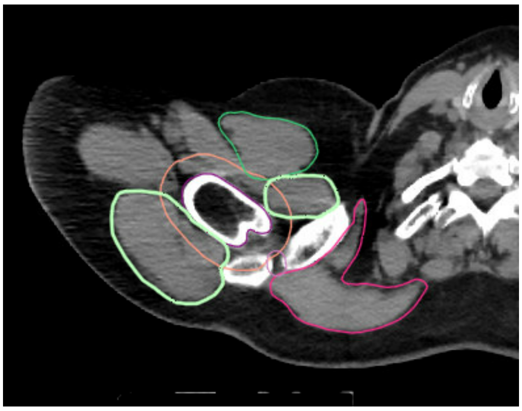

Seen in the first image below is the final slice of the anterior deltoid before its origin at the clavicle. The second image shows one of the last slices of the posterior deltoid, just past its origin at the spine of the scapula.

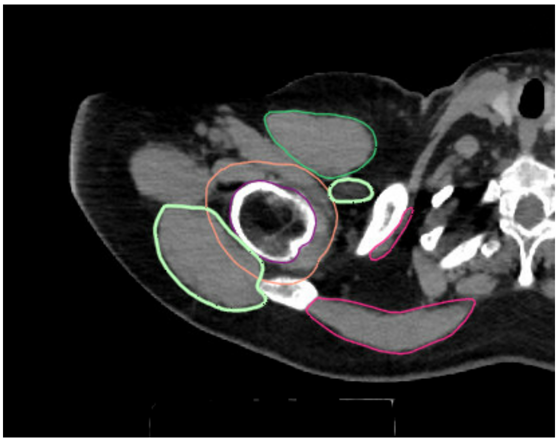
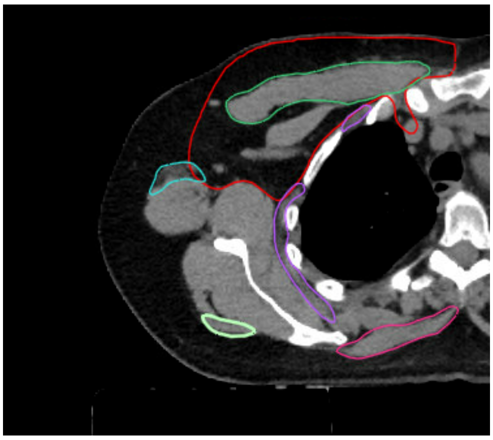

3) Glenohumeral Joint (opaque blue in all images): utilize the bone window to best visualize the joint space. As with the AC joint, use a 1 cm brush to contour the space between the humerus and the scapula (glenoid fossa).

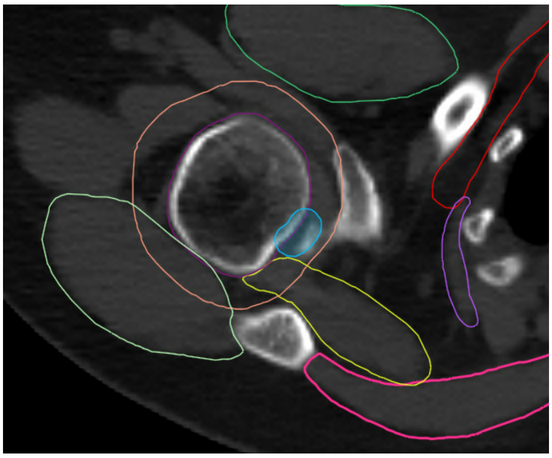
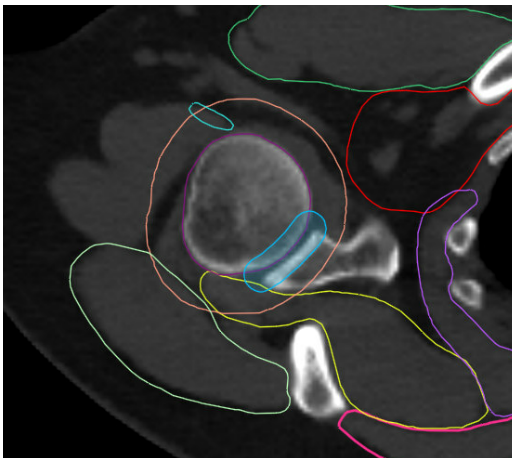

4) Humeral Head (dark purple in all images): Utilize the bone window to contour this structure. Superiorly, this contour will start approximately 5 cm distal to the medial humeral head, along a line parallel to the shaft of the humerus, as seen below on the first sagittal image. This contour ends when the humeral head disappears.

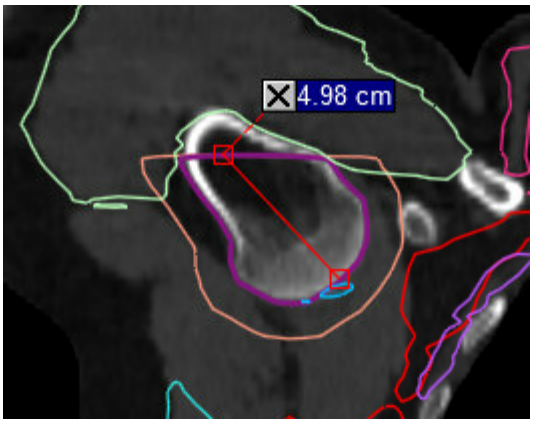

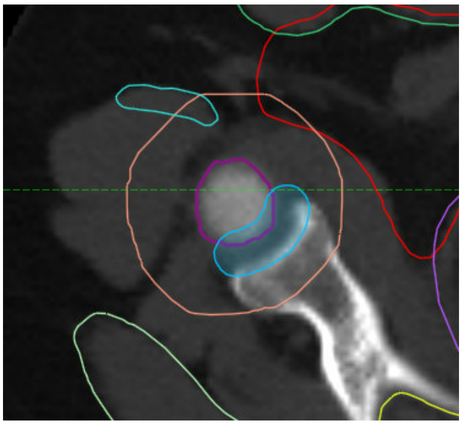

5) Latissimus Dorsi (cyan/blue in all images): The latissimus is a large muscle originating from the spinous processes of thoracic vertebrae 7-12, the thoracolumbar fascia, and the iliac crest. It inserts on the intertubercular groove of the humerus. Superiorly, this structure is very small and adjacent to the humerus at its insertion. Moving inferiorly, it is the anterior-most muscular structure in the second image, just anterior to the teres major (yellow arrow).

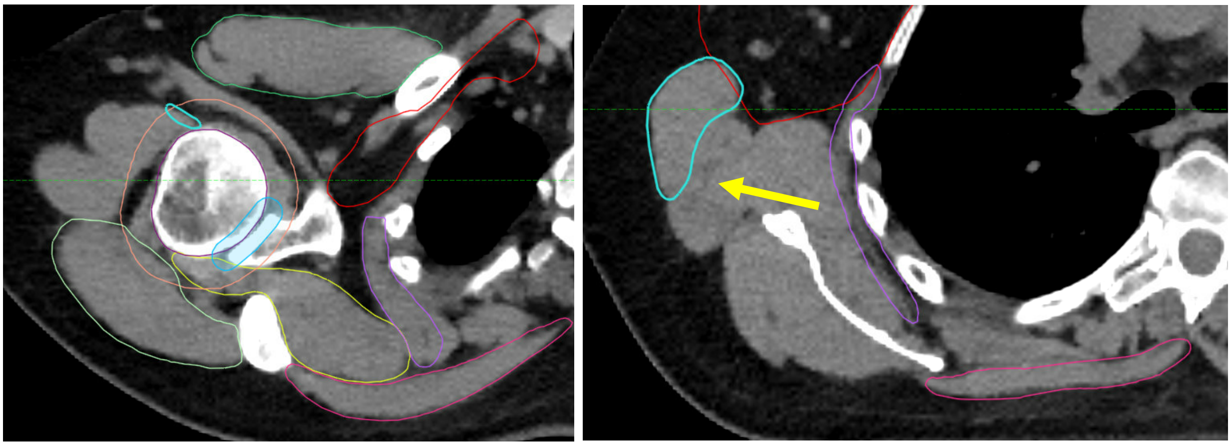

The latissimus begins to broaden as the contour progresses more inferiorly. Eventually the origin along the midline becomes apparent and two structures are contoured. These structures will join more inferiorly, and the contour continues until 3 cm inferior to the CTV.

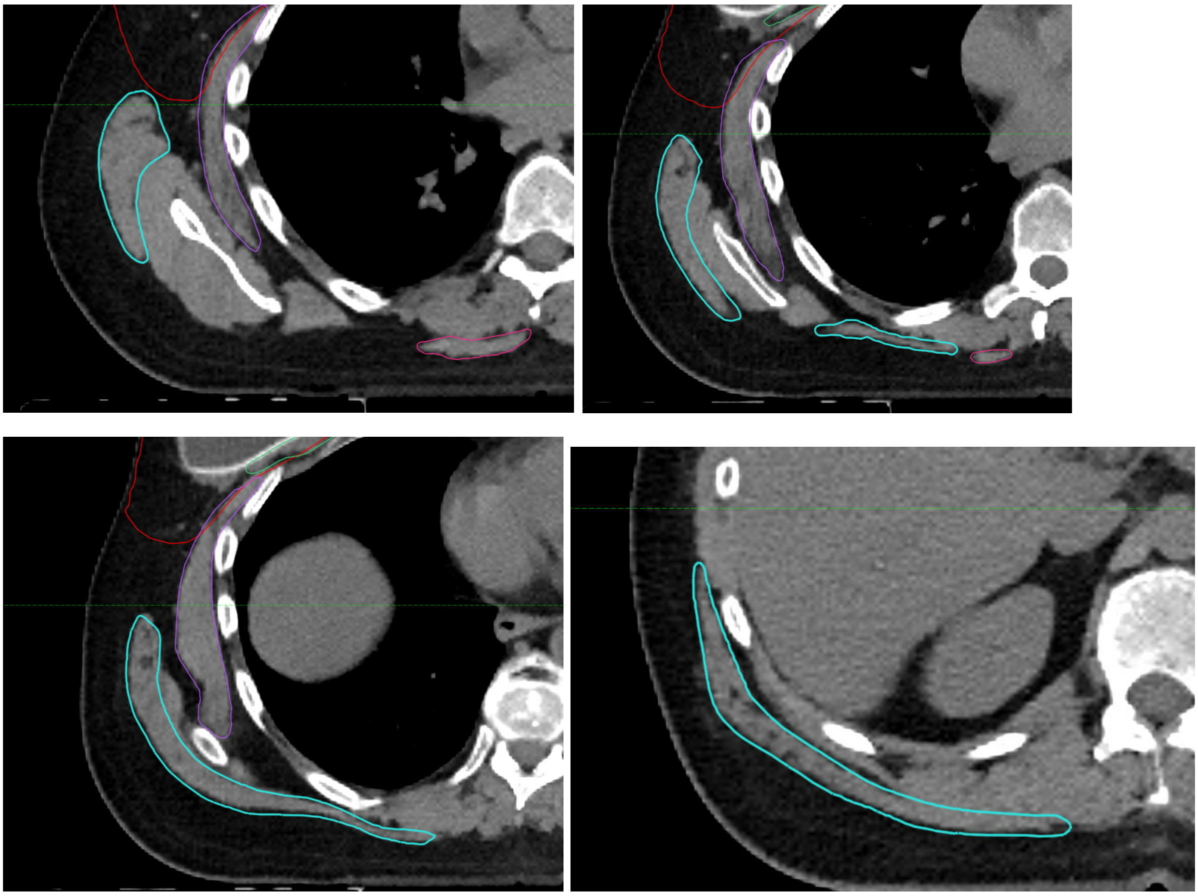

6) Pectoralis Major (kelly green in all images): With arms up, the pectoralis major will first be visible adjacent to the humerus and deltoid at its insertion at the intertubercular sulcus of the humerus.

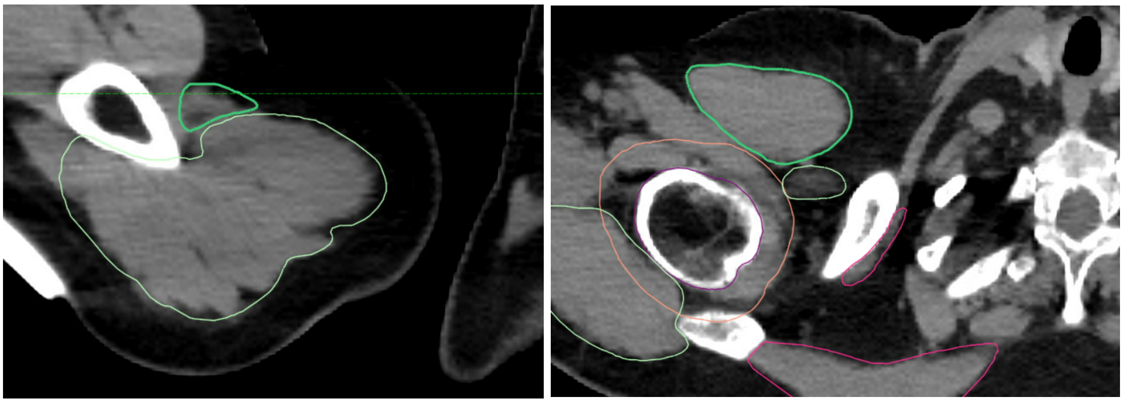

More inferiorly, the muscle continues (distinct from the posterior pectoralis minor, light pink arrow in the second image below), until its origin at the clavicle (first image) and sternum (second image). The sternal insertion often has multiple segments, and the pectoralis major continues inferiorly for many centimeters before disappearing completely. In the more inferior images, note the adjacent serratus anterior in purple (purple arrow).

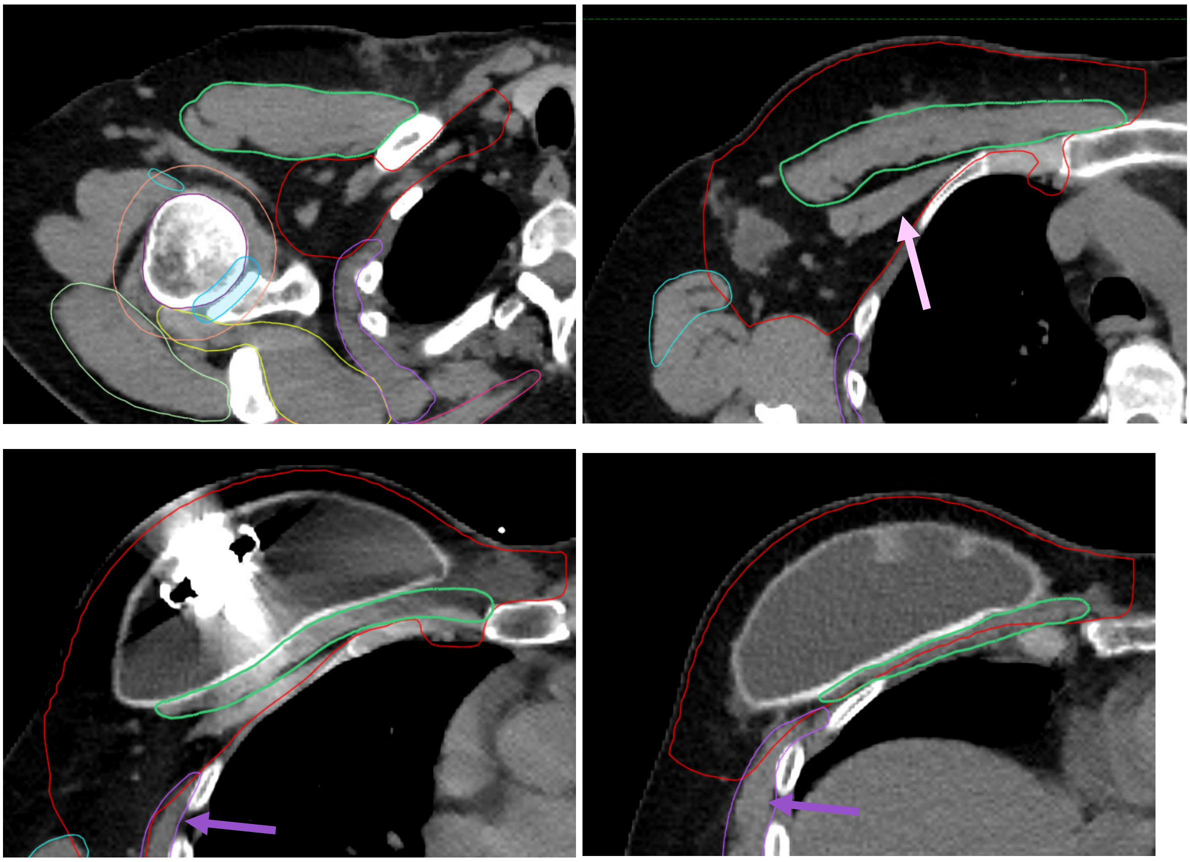

7) Rotator Cuff Attachments (orange in all images): Create this structure by expanding the humeral head contour by 1 cm. Then, crop all slices distal to the humeral head contour (this is the superior direction if the patient's arms are up – as seen in the second image below).

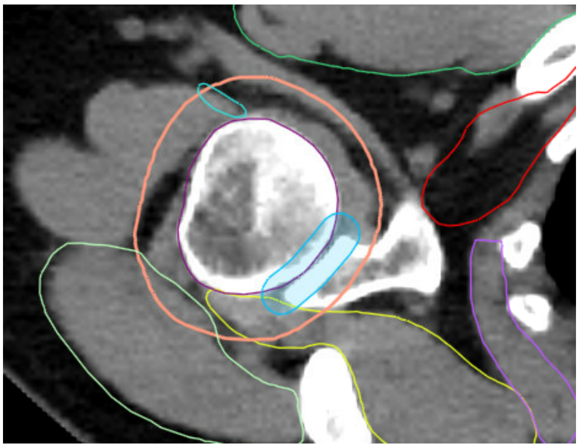
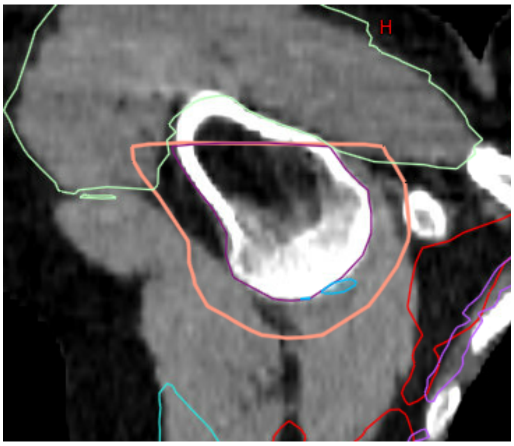

This contour reliably captures the humeral insertions of the supraspinatus, infraspinatus, teres minor, and subscapularis. Each of these four muscle insertions can be seen below. Although individual rotator cuff muscles were not contoured for the purposes of dose comparison, they are contoured here to demonstrate that the rotator cuff attachment structure reliably captures these insertion points. In the sagittal images below, the rotator cuff attachment structure is seen in orange and the humeral head is purple. The supraspinatus is yellow, the infraspinatus dark blue, the teres minor light blue, and the subscapularis green.

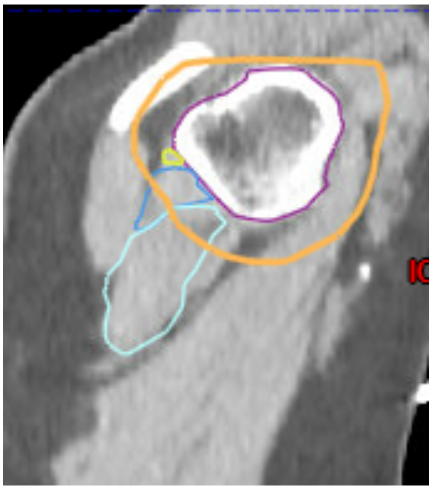
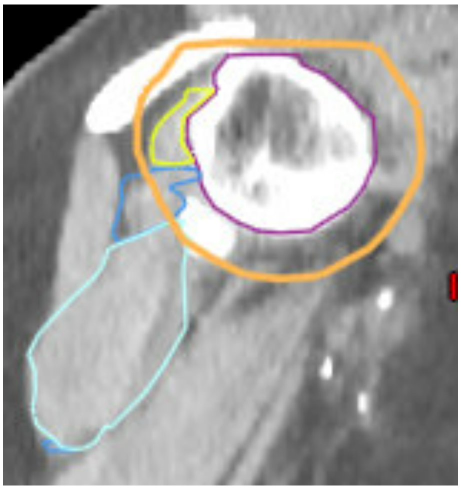

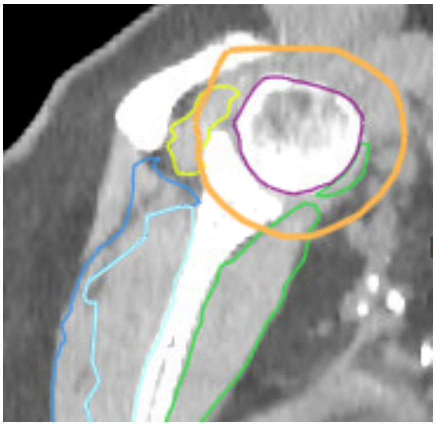
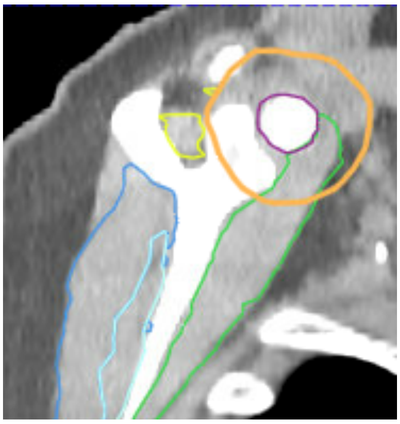

8) Serratus Anterior (purple in all images): The serratus anterior is a fan-shaped muscular structure which is sometimes discontiguous (in the axial view). It fans from the lateral surfaces of the first 8-9 ribs to the scapula. It first appears just lateral to the 2nd and 3rd rib for this patient, and scrolling inferiorly, we see the muscle body become larger and associate with more ribs as well as the scapula posteriorly.

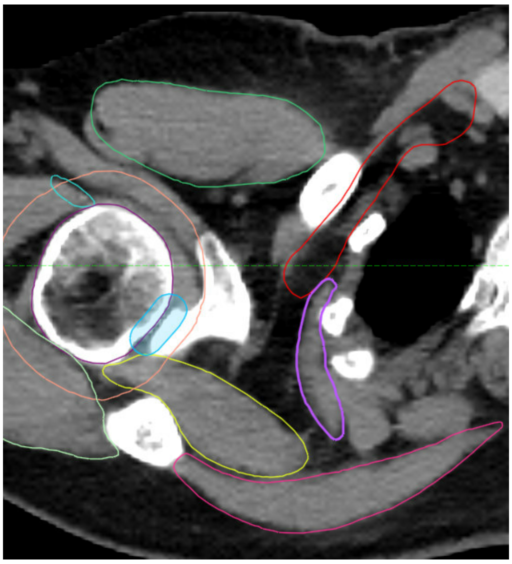

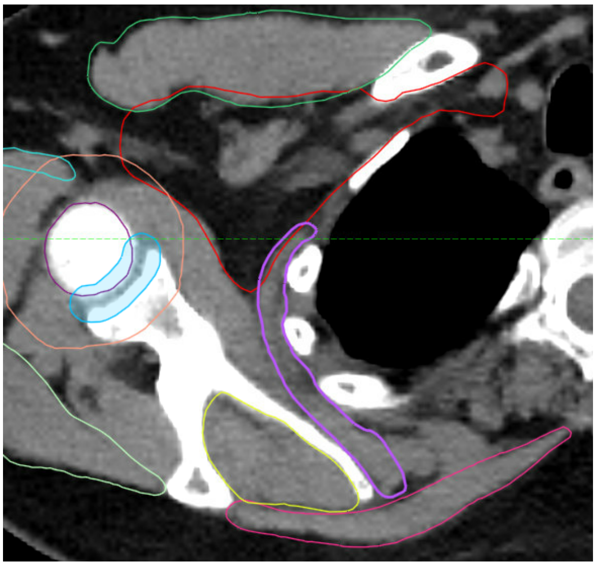

It may at times become discontiguous as the “fanning” of the serratus anterior takes place, as seen in the first axial image. This fanning shape is more evident in the sagittal view, seen in the third and fourth images below.

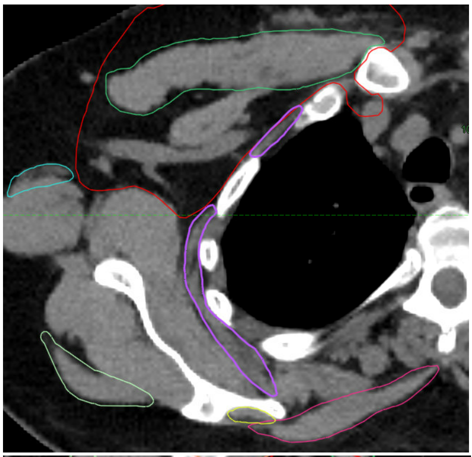

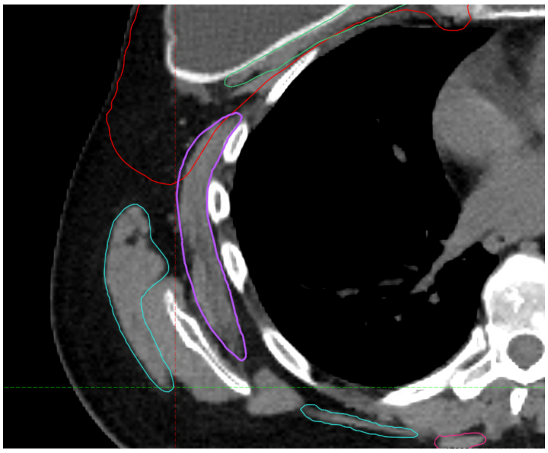

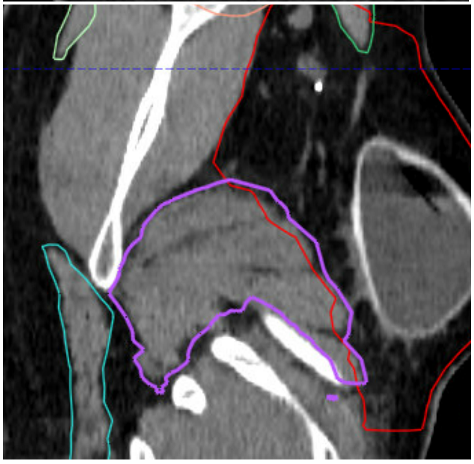

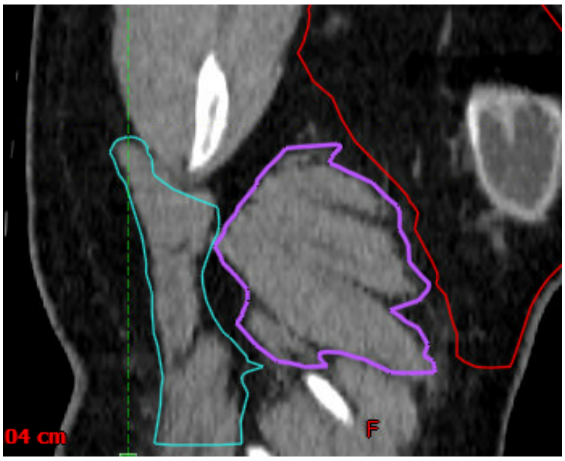

The serratus is just lateral to the pectoralis major (green arrow below) moving inferiorly, until it becomes smaller and then disappears.

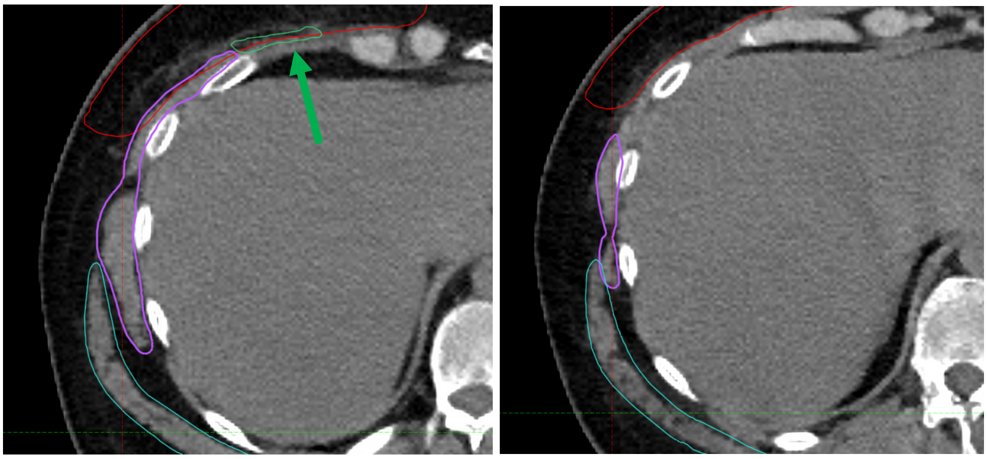

9) Supraspinatus (yellow in all images): The supraspinatus originates at the scapula and inserts on the greater tubercle of the humerus.

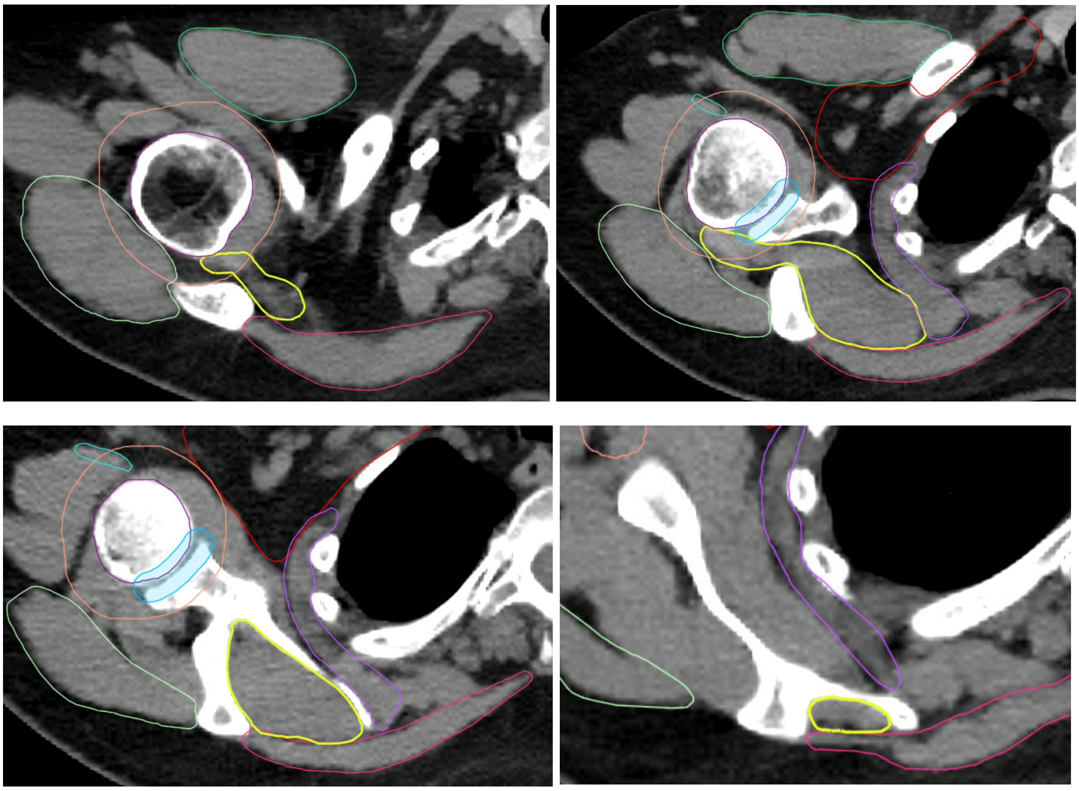

10) Trapezius (bright pink in all images): The trapezius is a superficial diamond-shaped muscle which originates from the skull, nuchal ligament, and spinous processes of C7-T12. Portions of the trapezius insert on the clavicle, acromion, and scapular spine. Contour all parts together as one structure. Start the trapezius contour superiorly at the superior aspect of the C5 vertebrael body. Continue inferiorly until it disappears. The superior-most portion of the contoured trapezius is seen below in bright pink:

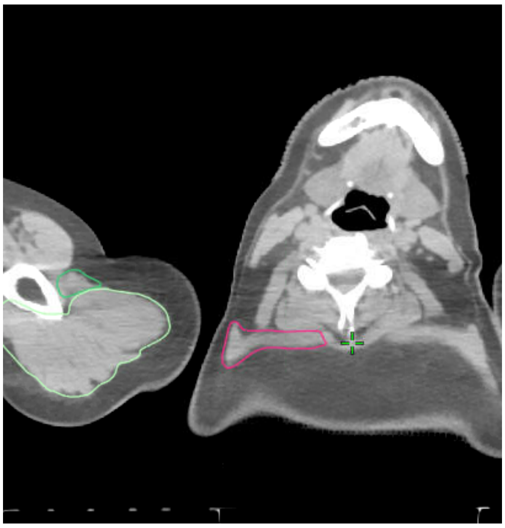
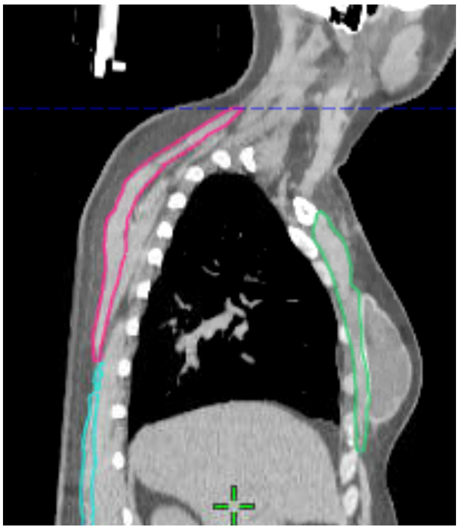

The scapular and clavicle attachments are seen here on these sagittal and axial images as contouring continues inferiorly:

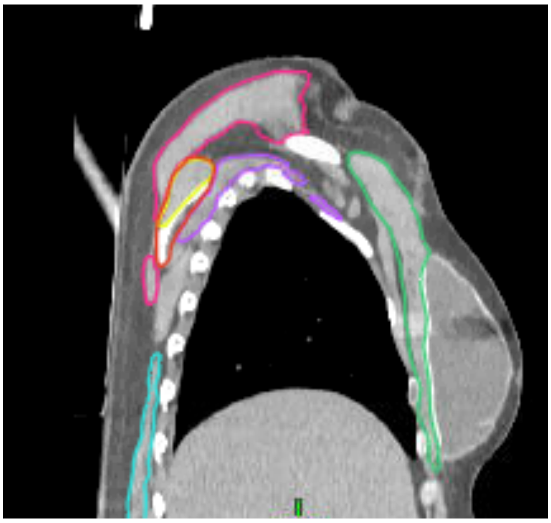
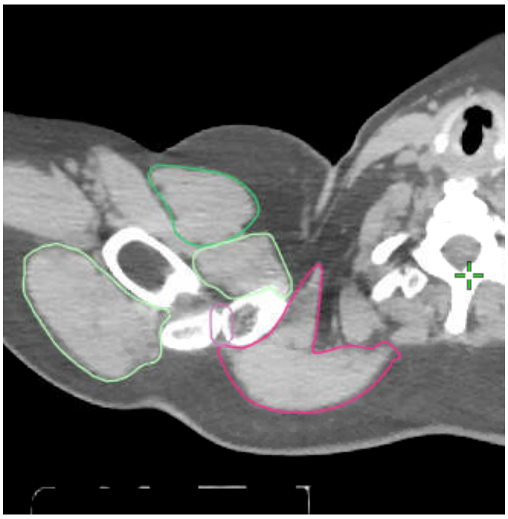

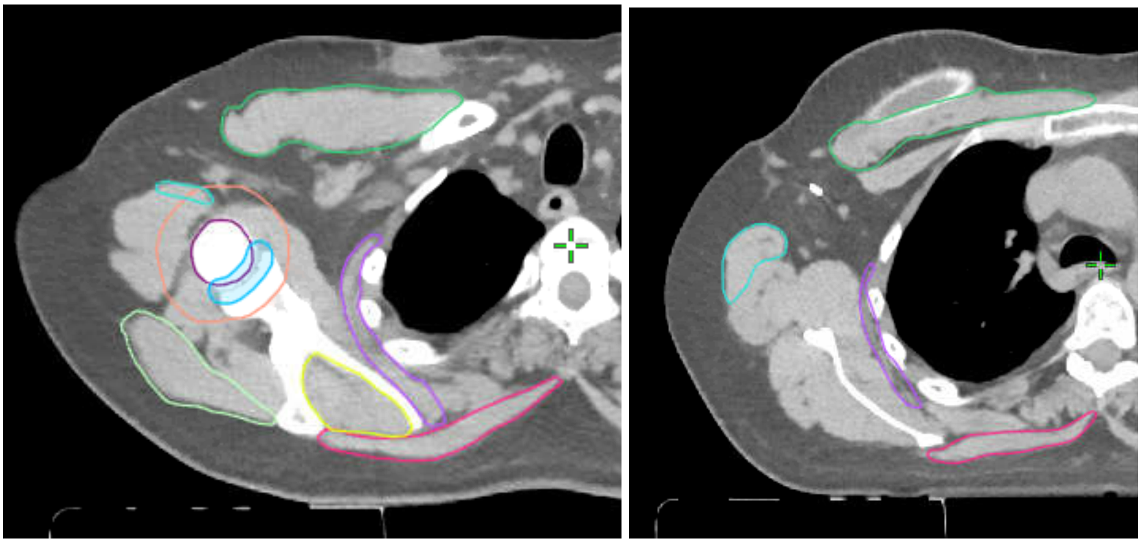

Continue contouring inferiorly until the trapezius disappears. This may be superior to T12 for some patients.

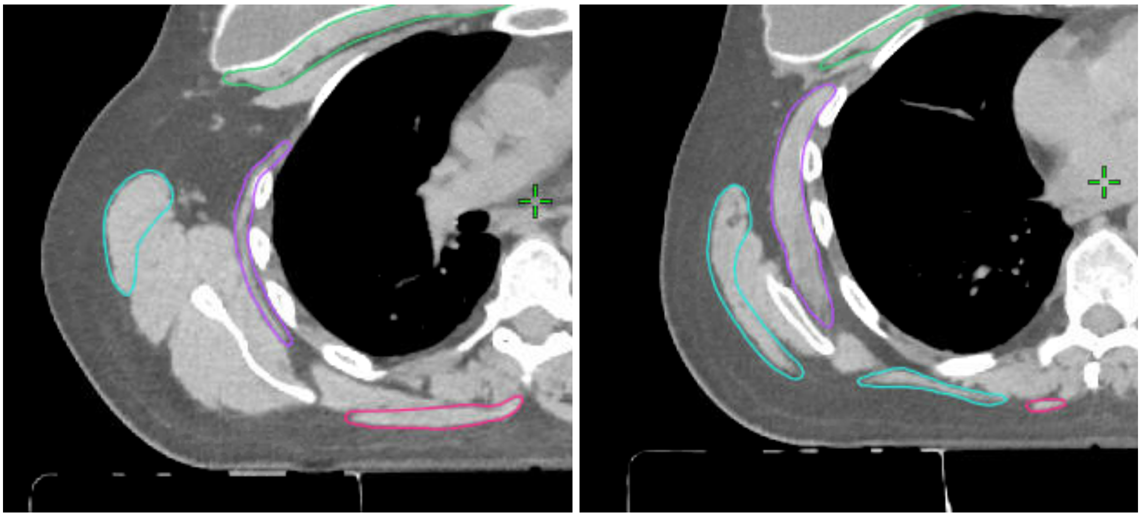

Additional analysis: 3D plans, optimized VMAT plans

Supplemental Table 1: Wilcoxon signed rank sum tests comparing the 15 patients with IMPT, VMAT (un-optimized), and 3D radiation plans

**Structure**	**Metric**	**Mean IMPT (n=15)**	**Mean VMAT (n=15)**	**Mean 3D (n=15)**	**Mean Difference 3D - IMPT**	**p-value**	**Mean Difference 3D - VMAT**	**p-value**
Deltoid	D5 cc	7.3 Gy	24.2 Gy	32.3 Gy	25.0 Gy	<0.001*	8.1 Gy	0.026
	D10 cc	2.1 Gy	21.2 Gy	22.7 Gy	20.6 Gy	<0.001*	1.6 Gy	0.679
	D50 cc	0.2 Gy	7.5 Gy	1.5 Gy	1.2 Gy	0.002*	-6.0 Gy	<0.001*
Latissimus Dorsi	D5 cc	44.0 Gy	48.5 Gy	53.1 Gy	9.1 Gy	<0.001*	4.6 Gy	0.001*
	D10 cc	38.1 Gy	45.3 Gy	52.2 Gy	14.1 Gy	<0.001*	6.9 Gy	<0.001*
	D50 cc	6.4 Gy	24.8 Gy	15.9 Gy	9.5 Gy	0.679	-8.9 Gy	0.135
Serratus Anterior	D5 cc	49.2 Gy	53.5 Gy	53.5 Gy	4.3 Gy	<0.001*	-0.1 Gy	0.389
	D10 cc	46.7 Gy	52.1 Gy	52.4 Gy	5.7 Gy	<0.001*	0.3 Gy	0.421
	D50 cc	20.1 Gy	35.2 Gy	30.1 Gy	10.0 Gy	0.005	-5.1 Gy	0.151
Supraspinatus	D5 cc	2.9 Gy	25.9 Gy	43.4 Gy	40.5 Gy	<0.001*	17.5 Gy	<0.001*
	D10 cc	1.8 Gy	22.5 Gy	42.7 Gy	40.9 Gy	<0.001*	20.1 Gy	<0.001*
Trapezius	D5 cc	18.1 Gy	28.6 Gy	44.4 Gy	26.3 Gy	<0.001*	15.8 Gy	<0.001*
	D10 cc	11.0 Gy	23.2 Gy	43.3 Gy	32.3 Gy	<0.001*	20.2 Gy	<0.001*
	D50 cc	0.8 Gy	12.1 Gy	29.7 Gy	28.9 Gy	<0.001*	17.6 Gy	<0.001*
Pectoralis Major	D5cc	52.7 Gy	55.5 Gy	55.7 Gy	3.0 Gy	<0.001*	0.1 Gy	1
	D10cc	52.1 Gy	55.0 Gy	55.0 Gy	2.9 Gy	<0.001*	-0.1 Gy	0.454
	D50cc	49.3 Gy	52.1 Gy	52.1 Gy	2.9 Gy	<0.001*	0.1 Gy	1
Acromioclavicular Joint	Mean	0.5 Gy	9.4 Gy	2.6 Gy	2.1 Gy	<0.001*	-6.8 Gy	0.002*
	V15 Gy	0.0%	21.8%	2.5%	2.5 Gy	0.063	-19.3%	0.083
	V30 Gy	0.0%	0.0%	0.2%	0.2 Gy	0.125	0.2%	0.125
Glenohumeral Joint	Mean	5.3 Gy	27.0 Gy	22.9 Gy	17.6 Gy	<0.001*	-4.1 Gy	0.151
	V15 Gy	10.5%	93.3%	58.9%	48.4%	<0.001*	-34.4%	<0.001*
	V30 Gy	0.5%	37.8%	36.3%	35.8%	<0.001*	-1.5%	0.890
Humeral Head	Mean	2.8 Gy	15.5 Gy	5.9 Gy	3.1 Gy	0.010	-9.5 Gy	<0.001*
	V15 Gy	4.7%	45.2%	10.2%	5.5%	0.330	-35.0%	<0.001*
	V30 Gy	0.2%	9.6%	6.5%	6.3%	0.003	-3.0%	0.542
Rotator Cuff Attachments	Mean	6.0 Gy	18.2 Gy	10.2 Gy	4.3 Gy	0.001*	-7.9 Gy	<0.001*
	V15 Gy	14.7%	51.9%	21.9%	7.2%	0.023	-30.0%	<0.001*
	V30 Gy	6.4%	18.5%	15.8%	9.4%	<0.001*	-2.7%	0.330

* indicates statistical significance at an alpha = 0.0172 given the Bonferroni correction for multiple comparisons. Note that negative values indicate that the 3D radiation plan on average delivered lower doses than its comparator plan (either IMPT or VMAT).
